# Burden of postmenopausal breast cancer attributable to excess body weight: comparative study of body mass index and CUN-BAE in MCC-Spain study

**DOI:** 10.1136/jech-2023-220706

**Published:** 2024-10-17

**Authors:** Naiara Cubelos-Fernández, Verónica Dávila-Batista, Tania Fernández-Villa, Gemma Castaño-Vinyals, Beatriz Perez-Gomez, Pilar Amiano, Eva Ardanaz, Irene Delgado Sillero, Javier Llorca, Guillermo Fernández Tardón, Juan Alguacil, Mercedes Vanaclocha Espí, Rafael Marcos-Gragera, Víctor Moreno, Nuria Aragones, Ane Dorronsoro, Marcela Guevara, Sofía Reguero Celada, Marina Pollan, Manolis Kogevinas, Vicente Martín

**Affiliations:** 1 Gene–Environment Interactions and Health Research Group (GIIGAS), University of León Institute of Biomedicine, Leon, Spain; 2 Gerencia de Atencion Primaria, Área de Salud de Valladolid, Valladolid, Spain; 3 Consortium for Biomedical Research in Epidemiology and Public Health, CIBERESP, Madrid, Spain; 4 Research Institute of Biomedical and Health Sciences, University of Las Palmas de Gran Canaria, Las Palmas de Gran Canaria, Spain; 5 Gene–Environment Interactions and Health Research Group (GIIGAS), Institute of Biomedicine (IBIOMED), University of León, Leon, Spain; 6 Barcelona Institute for Global Health, ISGlobal, Barcelona, Spain; 7 Hospital del Mar Medical Research Institute, Barcelona, Spain; 8 Universitat Pompeu Fabra, Barcelona, Spain; 9 Department of Chronic Disease Epidemiology, National Centre of Epidemiology, Instituto de Salud Carlos III, Madrid, Spain; 10 Sub Directorate for Public Health and Addictions of Gipuzkoa, Ministry of Health of the Basque Government, Donostia-san Sebastian, Spain; 11 Epidemiology of Chronic and Communicable Diseases Group, Biodonostia Health Research Institute, Donostia-san Sebastian, Spain; 12 Instituto de Salud Pública y Laboral de Navarra, Instituto de Investigación Sanitaria de Navarra (IdiSNA), Pamplona, Spain; 13 Universidad de Cantabria, Santander, Spain; 14 ISPA, Health Research Institute of the Principality of Asturias, Oviedo, Spain; 15 Natural Resources, Health and Environment Research Centre (RENSMA), Universidad de Huelva-Campus El Carmen, Huelva, Spain; 16 Cancer and Public Health, FISABIO, Valencia, Spain; 17 Epidemiology Unit and Girona Cancer Registry, Oncology Coordination Plan, Department of Health, Autonomous Government of Catalonia, Catalan Institute of Oncology Girona, University of Girona, Girona, Spain; 18 Descriptive Epidemiology, Genetics and Cancer Prevention Group, Institute of Biomedical Research of Girona Dr Josep Trueta (IDIBGI-CERCA), Girona, Spain; 19 Oncology Data Analytics Program, Catalan Institute of Oncology (ICO), L'Hospitalet del Llobregat, Barcelona, Spain; 20 Colorectal Cancer Group, ONCOBELL Program, Institut d’Investigació Biomèdica de Bellvitge (IDIBELL), L'Hospitalet de Llobregat, Barcelona, Spain; 21 Department of Clinical Sciences, Faculty of Medicine and health Sciences and Universitat de Barcelona Institute of Complex Systems (UBICS), University of Barcelona (UB), L’Hospitalet de Llobregat, Barcelona, Spain; 22 Public Health Division, Department of Health of Madrid, Madrid, Spain; 23 Sub Directorate for Public Health and Addictions of Gipuzkoa, Ministry of Health of the Basque Government, Donosti-San Sebastian, Spain; 24 Gerencia de Atencion Primaria, Área de Salud de León, Leon, Spain

**Keywords:** BREAST NEOPLASMS, OBESITY, HEALTH IMPACT ASSESSMENT

## Abstract

**Background:**

10% of postmenopausal breast cancer cases are attributed to a high body mass index (BMI). BMI underestimates body fat, particularly in older women, and therefore the cancer burden attributable to obesity may be even higher. However, this is not clear. CUN-BAE (Clínica Universidad de Navarra–Body Adiposity Estimator) is an accurate validated estimator of body fat, taking into account sex and age. The objective of this study was to compare the burden of postmenopausal breast cancer attributable to excess body fat calculated using BMI and CUN-BAE.

**Methods:**

This case–control study included 1033 cases of breast cancer and 1143 postmenopausal population controls from the multicase–control MCC-Spain study. Logistic regression models were used to calculate odds ratios (ORs). The population attributable fraction (PAF) of excess weight related to breast cancer was estimated with both anthropometric measures. Stratified analyses were carried out for hormone receptor type.

**Results:**

Excess body weight attributable to the risk of breast cancer was 23.0% when assessed using a BMI value ≥30 kg/m^2^ and 38.0% when assessed using a CUN-BAE value of ≥40% body fat. Hormone receptor stratification showed that these differences in PAFs were only observed in hormone receptor positive cases, with an estimated burden of 19.9% for BMI and 41.9% for CUN-BAE.

**Conclusion:**

These findings suggest that the significance of excess body fat in postmenopausal hormone receptor positive breast cancer could be underestimated when assessed using only BMI. Accurate estimation of the cancer burden attributable to obesity is crucial for planning effective prevention initiatives.

**WHAT IS ALREADY KNOWN ON THIS TOPIC**

- Obesity is a well known risk factor for postmenopausal breast cancer, and body mass index (BMI) is the most commonly used measure.
- However, BMI underestimates body fat in older women, leading to underestimation of the cancer burden attributable to obesity.
- It is important to compare the cancer burden attributable to obesity calculated using BMI with more accurate measures of body fat.

**WHAT THIS STUDY ADDS**

- Our study estimated that 38% of incident postmenopausal breast cancer cases in Spain might be attributable to high body weight.
- The burden of postmenopausal hormone receptor positive breast cancer attributable to excess body weight was higher when assessed with the Clínica Universidad de Navarra–Body Adiposity Estimator (CUN-BAE) than with BMI.

**HOW THIS STUDY MIGHT AFFECT RESEARCH, PRACTICE OR POLICY**

- The findings of this study highlight the importance of considering more accurate measures of body fat than BMI to estimate the cancer burden attributable to obesity in postmenopausal breast cancer.
- This information could potentially influence the planning of effective cancer prevention initiatives.

## Introduction

 Breast cancer is the most common cancer among women, with an estimated >2.2 million new cases worldwide (46.8 per 100 000 persons incident) in 2022.^1^ Body mass index (BMI) is a well established risk factor for postmenopausal breast cancer.^2^ It is estimated that about 10% of cases can be due to a BMI >24.9 kg/m^2^.^3^ Most studies that have assessed the relationship between body fat and breast cancer were based on calculations of BMI.^3^,^6^

However, it is widely known that the correlation between body fat and BMI is not linear because it is influenced by several factors, such as sex, age and race. Therefore, BMI tends to underestimate the percentage of body fat, particularly in older women.^7 8^ Several alternative anthropometric measures have been proposed, either independently or in addition to BMI.^9^,^11^ One of these more accurate measures of body fat is CUN-BAE (Clínica Universidad de Navarra–Body Adiposity Estimator), a body fat estimator developed in a white population by Gómez-Ambrosi *et al*.^12^ This estimator uses BMI values, sex and age, and correlates better with body fat and metabolic disorders than BMI.^13^,^16^

Hormonal factors have an important role in the relationship between body weight and breast cancer. Obesity is a well established risk factor for oestrogen receptor positive breast cancer, because the production of oestrogen in fatty breast tissue is a key factor in tumour development.^4 6 17^ Nevertheless, previous studies have not evaluated the impact of body fat on the burden of breast cancer according to different types of tumour receptors. It is essential to determine the impact of excess fat on the risk of breast cancer to justify interventions aiming to prevent and control obesity, particularly in postmenopausal women.^18^,^20^

The aim of this study was to compare the burden of breast cancer cases that can be attributed to excess body fat in postmenopausal women, calculated with BMI and CUN-BAE, and to examine this relation in terms of different tumour receptors.

## Materials and methods

### Study design and participants

MCC-Spain is a population-based multicenter case-control study, carried out in September 2008 to December 2013 in 13 Spanish provinces (Asturias, Barcelona, Cantabria, Girona, Granada, Guipúzcoa, Huelva, León, Madrid, Murcia, Navarra and Valencia). The overall objective of the study was to evaluate the environmental and genetic factors associated with colorectal cancer, breast cancer, gastric cancer, prostate cancer and chronic lymphocytic leukaemia.^21^

Inclusion criteria were participants aged 20–85 years, residenccy in the recruitment area for at least 6 months before recruitment and physically/mentally capable of answering the epidemiological survey. Recruitment of cases and controls was carried out simultaneously. Only incident breast cancer cases confirmed histologically in the 23 collaborating hospitals were included. The population controls were selected randomly and matched by age, sex and region to the cases. Controls were invited to participate, after being selected from the administrative registers of the primary care centres located within each hospital’s catchment area.^21^ MCC-Spain includes 1738 breast cancer cases and 1910 matched population controls. More details on the project can be found at https://www.mccspain.org.

**Figure 1:**
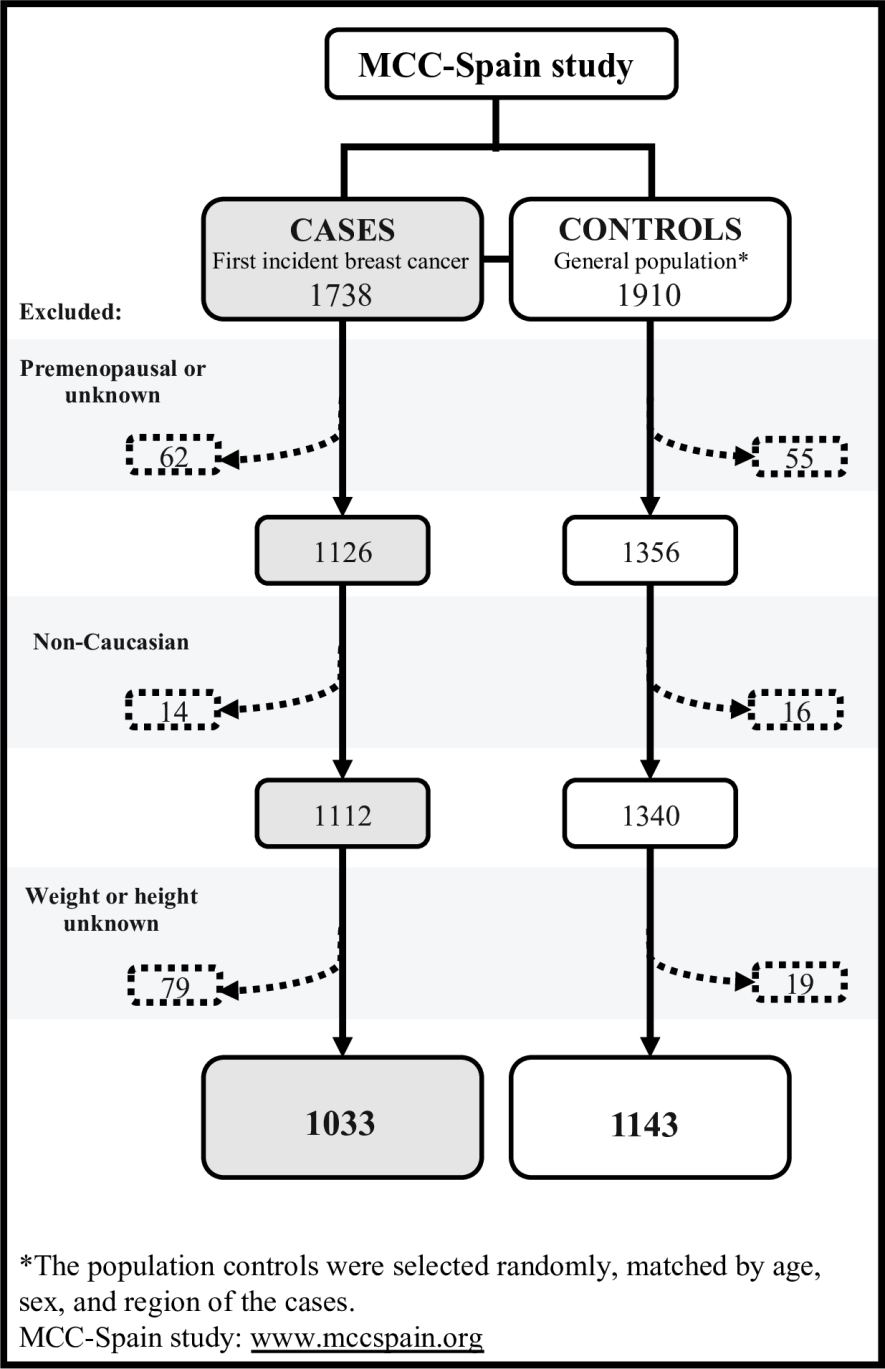
Flowchart of the selection process of breast cancer cases in postmenopausal women, and in population controls, in the MCC-Spain study.

This study included postmenopausal white women with available anthropometric information. The final sample included 1033 breast cancer cases and 1143 controls. Figure 1 shows the flowchart of the selection process. Non-white participants were excluded because BMI cut-off points differ in other races, and CUN-BAE has been validated only in white populations.

### Data collection

Data on sociodemographic factors, lifestyle, personal/family medical history and reproductive history were collected by a computerised structured epidemiological questionnaire, administered by trained personnel in a face-to-face interview.^21^ Dietary information was obtained with a validated semi-structured 140 item food frequency questionnaire,^22^ which assessed dietary intake in the previous year. This questionnaire was provided to and self-administered by participants after the first interview (response rate 88%). Also, information about past alcohol consumption (for ages 30–40 years) was collected mainly through a self-administered questionnaire. Clinicopathological information on the cancer cases was obtained and validated from medical records, including the hormone receptor status of the tumour, degree of differentiation and histological type.

### Definition of breast cancer cases

The main outcome variable was incident breast cancer confirmed histologically. The International Classification of Diseases, 10th revision codes used were C50 (invasive breast cancer), D05.1 and D05.7 (breast cancer in situ). The subtype of breast cancer was classified according to the pressence of positive hormone receptors (HR+=oestrogen positive or progesterone positive) or positive human epidermal growth factor (Erb2+), irrespective of oestrogen or progesterone status, or triple negative tumours (HR−=none of the three receptors expressed).^12^

### Anthropometric measures

In face-to-face interviews carried out by trained personnel, participants provided data on weight and height. BMI of the cancer cases was obtained 1 year before diagnosis and BMI for controls was measured at the time of the interview. BMI was calculated as the ratio of weight (kg) to height squared (kg/m^2^) and was categorised using the WHO standards^23^: <25; 25–29.9; 30–34.9; and ≥35 kg/m^2^.

CUN-BAE was calculated with the equation developed by Gómez-Ambrosi *et al*^12^: % BF=−44.988+(0.503×age)+(10 689×sex)+(3.172×BMI)–(0.026×BMI^2^+(0.181×BMI×sex)–(0.02×BMI×age)–(0.005×BMI^2^×sex)+(0.00021×BMI^2^×age), where age is expressed in years and sex is encoded as men=0 and women=1. CUN-BAE was categorised according to the estimated percentage of body fat: <35%, 35–39.9%, 40–44.9% and ≥45%.

### Statistical analysis

A descriptive analysis of the characteristics of participant was carried out using arithmetic mean (SD) for quantitative variables, and absolute and relative frequencies (%) for categorical variables. To test differences in general characteristics between cases and controls, the χ^2^ test was used.

Odds ratios (ORs) were estimated with 95% CIs for both measures (BMI and CUN-BAE) for breast cancer cases using unconditional logistic regression. The multivariate model included the following covariates: age at recruitment (years) ≤50, 51–60, 61–70 or >70 years; age of menarche <12, 12–13, ≥14 years or unknown; nulliparity (number of children) none, 1, 2, ≥3 or unknown; breastfeeding time (months) none, <6, ≥6 or unknown; energy intake (calories/day) <1500, 1500–2000, ≥2000 or unknown; family history of breast cancer no or yes; socioeconomic status low, medium or high; alcohol consumption (past or present g/day) 0, <12, ≥12 or unknown; smoking status never, yes or former; physical activity (METS×hours/week for mean year) 0, <8, 8–16, >16 or unknown; anti-inflammatory drug use never, some or unknown; oral contraceptive treatment never, some or unknown; and oral supplementary hormone treatment never, some, unknown.

A priori modifying effect by hormone receptor type in breast cancer was conducted, stratifying by tumour receptor status: hormone receptor positive (oestrogen or progesterone or both receptors), Erb2+ and triple negative.

Finally, the population attributable fraction (PAF) with 95% CIs was estimated as an epidemiological measure to assess the impact of both exposures (BMI vs CUN-BAE) on the burden of breast cancer in our Spanish population. PAFs were calculated in the MCC-Spain study datasets using the formula for exposures with k+1 levels:

PAF=1−(Σ(pr/OR)), where *pr* is the proportion of cases in each body fat category and *OR* is the adjusted OR for each category.^24^ All analyses were performed with the statistical software package Stata MP V.15 (StataCorp). Statistical significance was set at a two sided p value of <0.05.

## Results

We included 1033 incident postmenopausal breast cancer cases and 1143 population controls from the MCC-Spain study. Table 1 shows the characteristics of participants. Compared with controls, breast cancer cases presented at a lower mean age, had a smaller number of children and higher energy intake. Cases more often had a first degree family history of breast cancer and low socioeconomic status. Mean BMI was 26.3 (SD 4.8) kg/m^2^ in controls and 27.2 (4.9) kg/m^2^ in cases. Mean CUN-BAE was 39.7 (5.5)% in controls and 40.4 (5.5)% in cases.

**Table 1:** Descriptive characteristics of study participants

Characteristics		Breast cancer cases(n=1033)		Controls(n=1143)		P value*
		n	%	n	%	
Age (years)	<50	92	8.9	71	6.2	<0.001
	51–60	373	36.1	345	30.2	
	61–70	355	34.4	400	35.0	
	>70	213	20.6	327	28.6	
Menarche age (years)	<12	211	20.4	221	19.3	0.068
	12–13	472	45.7	512	44.8	
	≥14	333	32.2	403	35.3	
	Unknown	17	1.7	7	0.6	
No of children	Never	182	17.6	174	15.2	0.020
	1	149	14.4	141	12.3	
	2	414	40.1	464	40.6	
	≥3	282	27.3	363	31.8	
	Unknown	6	0.6	1	0.1	
Breast feeding	Never	335	32.4	355	31.1	0.510
	<6 months	354	34.3	423	37.0	
	≥6 months	224	21.7	228	19.9	
	Unknown	120	11.6	137	12.0	
Total energy intake (calories/day)	<1500	273	26.4	348	30.4	0.134
	1500–2000	332	32.1	370	32.4	
	≥2000	268	25.9	267	23.4	
	Unknown	160	15.5	158	13.8	
Family history of breast cancer	No	877	85.7	1018	89.3	0.010
	Yes	146	14.3	122	10.7	
Socioeconomic status	Low	403	39.0	411	36.0	0.258
	Medium	506	49.0	576	50.4	
	High	124	12.0	156	13.6	
Alcohol intake (g/day)	0	240	23.2	277	24.2	0.190
	<12	487	47.1	574	50.2	
	≥12	146	14.1	134	11.7	
	Unknown	160	15.5	158	13.8	
Smoking status	Never	653	63.2	730	63.9	0.750
	Yes	380	36.8	413	36.1	
Physical activity (METS×hour/week)	0	407	39.4	424	37.1	0.015
	<8	165	16.0	192	16.8	
	8–16	127	12.3	152	13.3	
	>16	334	32.23	363	31.8	
	Unknown	0	0.0	12	1.0	
Anti-inflammatory drugs	Never	413	40.0	527	46.1	0.013
	Sometimes	581	56.2	572	50.0	
	Unknown	39	3.8	44	3.9	
Oral contraceptives	Never	631	61.1	657	57.5	0.010
	Sometime	397	38.4	486	42.5	
	Unknown	5	0.5	0	0.0	
Hormone replacement therapy	Never	883	45.5	962	84.2	0.350
	Sometime	110	10.6	122	10.7	
	Unknown	40	3.9	59	5.1	

p=0.05 was considered statistically significant.

* Differences between cases and controls were tested using the χ2 test.

**Figure 2:**
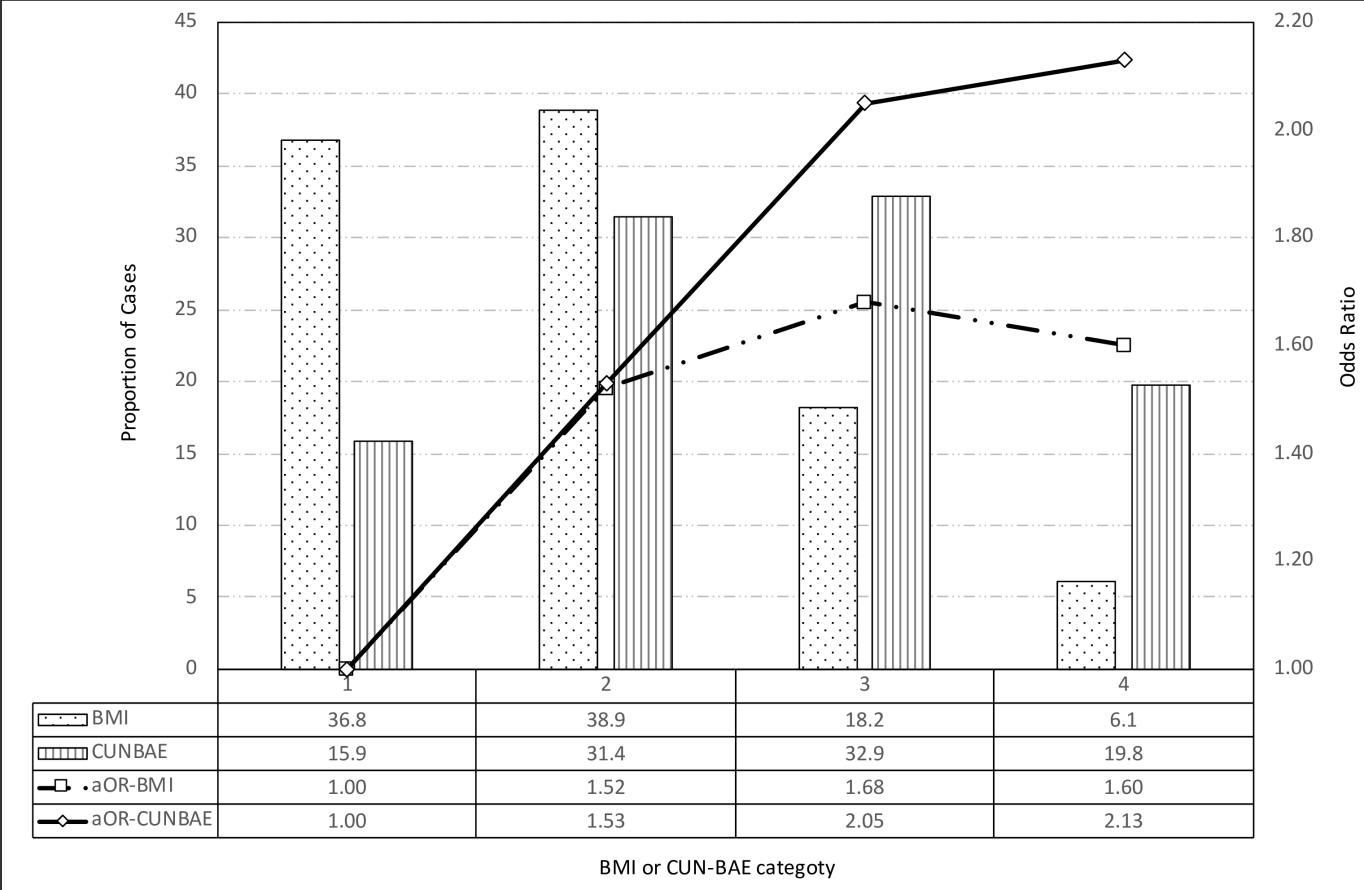
Prevalence of body fat, according to body mass index (BMI) and Clínica Universidad de Navarra–Body Adiposity Estimator (CUN-BAE) category, and ORs for postmenopausal breast cancer. BMI was classified as <25 (reference), 25–29.9, 30–34.9 and ≥35 kg/m^2^. CUN-BAE was classified as <35 (reference), 35–39.9, 40–44.9 and ≥45 percentage body fat. ORs were adjusted (aOR) for age at recruitment (years) ≤50, 51–60, 61–70 or >70 years; age of menarche <12, 12–13, ≥14 or unknown; nulliparity (number of children) none, 1, 2, ≥3 or unknown; breastfeeding time (months) no, <6, ≥ 6 or unknown; energy intake (calories/day) <1500, 1500 to 2000, ≥2000 or unknown; family history of breast cancer no or yes; socioeconomic status low, medium or high; alcohol consumption (past or present g/day) 0, <12, ≥12 or unknown; smoking status never, yes or former; physical activity (METS×hours/week for mean year) 0, <8, 8–16, >16 or unknown; anti-inflammatory drug use never, some or unknown; oral contraceptive treatment never, some or unknown; and oral supplementary hormonal treatment never, some or unknown.

Figure 2 shows the distribution of obesity index exposure by BMI and CUN-BAE category. BMI <25 kg/m^2^ (reference) was observed in 45.0% of controls and in 36.8% of breast cancer cases, whereas a BMI ≥30 kg/m^2^ was observed in 19.9% of controls and 24.3% of breast cancer cases. CUN-BAE <35% (reference) was observed in 20.6% of controls and in 15.9% of cases, and CUN-BAE ≥40% was observed in 46.3% of controls and in 52.7% of cases.

The highest categories of CUN-BAE showed an increase in the risk of postmenopausal breast cancer (OR 2.13 for body fat ≥45% compared with the reference category <35%). However, no similar trend was observed for BMI, because the gradient declined after a BMI of ≥35 kg/m^2^. Because of the different distributions of exposure together with the small increase in the estimated OR for BMI, we showed that the estimated PAFs were 23.0% (95% CI 12.2% to 31.4%) for BMI and 38.0% (95% CI 23.1% to 49.6%) for CUN-BAE, as presented in table 2.

**Table 2:** Burden of postmenopausal breast cancer due to body fat using body mass index (BMI) and Clínica Universidad de Navarra–Body Adiposity Estimator (CUN-BAE)

	Controls		Postmenopausal breast cancer cases					
	No	%	No	%	OR	95% CI for OR	PAF	95% CI for PAF
BMI								
<25	521	45.6	380	36.8	1		**0.23**	0.122 to 0.314
25–29.9	395	34.5	402	38.9	**1.52**	1.24 to 1.86		
30.0–34.9	168	14.7	188	18.2	**1.68**	1.30 to 2.19		
≥35	59	5.2	63	6.1	**1.6**	1.08 to 2.40		
CUN-BAE								
<35	235	20.6	164	15.9	1		**0.38**	0.231 to 0.496
35.0–39.9	379	33.2	324	31.4	**1.53**	1.17 to 2.01		
40.0–44.9	331	29.0	340	32.9	**2.05**	1.54 to 2.72		
≥45	198	17.3	205	19.8	**2.13**	1.54 to 2.93		

ORs were adjusted (aOR) for age at recruitment (years) ≤50, 51–60, 61–70 or >70 years; age of menarche <12, 12–13, ≥14 or unknown; nulliparity (number of children) none, 1, 2, ≥3 or unknown; breastfeeding time (months) no, <6, ≥ 6 or unknown; energy intake (calories/day) <1500, 1500 to 2000, ≥2000 or unknown; family history of breast cancer no or yes; socioeconomic status low, medium or high; alcohol consumption (past or present g/day) 0, <12, ≥12 or unknown; smoking status never, yes or former; physical activity (METS×hours/week for mean year) 0, <8, 8–16, >16 or unknown; anti-inflammatory drug use never, some or unknown; oral contraceptive treatment never, some or unknown; and oral supplementary hormonal treatment never, some or unknown.

Bold typeface indicates significance at p=0.05.

PAFspopulation attributable fractions

Estimated PAFs for BMI and CUN-BAE by breast cancer hormone receptor type are shown in table 3. For breast cancer cases with positive hormone receptors (n=680 cases), a higher BMI contributed to a PAF of 19.9% (95% CI 9.1% to 27.8%) while with CUN-BAE, PAF was 41.9% (95% CI 26.3% to 61.2%). PAFs for breast cancer cases with positive hormone receptors were similar to those for overall breast cancer cases. These similar trends in PAFs reflected differences in the distribution of BMI versus CUN-BAE categories, as well as differences in OR values. For Erb2+ (178 cases) or triple negative (79 cases) cases, the PAF burden was the same when estimating PAF using BMI or CUN-BAE (%PAF for Erb2+ 23.4% vs 24.6%; %PAF for triple negative breast cancer 23.0% vs 26.3%, with BMI and CUN-BAE, respectively).

**Table 3:** Fraction of postmenopausal breast cancer cases attributable to body fat using body mass index (BMI) and Clínica Universidad de Navarra–Body Adiposity Estimator (CUN-BAE), by hormone receptor type

	BMI				CUN-BAE			
	**<25**	**25–29.9**	**30–34.9**	**≥35**	**<35**	**35–39.9**	**40–44.9**	**≥45**
Controls								
No	521	395	168	59	235	379	331	198
%	45.6	34.5	14.7	5.2	20.5	33.2	29	17.3
Positive hormone receptors								
No	248	264	126	42	100	216	228	136
%	36.5	38.8	18.5	6.2	14.7	31.8	33.5	20.0
OR	1	**1.53**	**1.68**	**1.61**	1	**1.66**	**2.20**	**2.23**
OR (95% CI)	1	1.21 to 1.92	1.25 to 2.25	1.13 to 3.56	1	1.22 to 2.27	1.59 to 3.05	1.54 to 3.22
PAFs (95% CI)	**0.199** (0.091 to 0.278)				**0.419** (0.263 to 0.612)			
Erb2+								
No	68	75	27	8	35	56	59	28
%	38.2	42.1	15.2	4.5	19.7	31.5	33.1	15.7
OR	1	**1.75**	1.43	1.20	1	1.26	**1.73**	1.39
OR (95% CI)	1	1.20 to 2.56	0.86 to 2.39	0.52 to 2.74	1	0.77 to 2.05	1.04 to 2.88	0.76 to 2.55
PAFs (95% CI)	**0.234** (0.004 to 0.374)				**0.249** (0.000 to 0.473)			
Triple negative hormone receptors								
No	27	27	19	6	12	25	19	23
%	34.2	34.2	24	7.6	15.2	31.6	24.1	29.1
OR	1	1.25	**2.13**	1.84	1	1.33	1.09	**2.30**
OR (95% CI)	1	0.70 to 2.22	1.11 to 4.09	0.68 to 4.96	1	0.62 to 2.85	0.48 to 2.48	0.99 to 5.39
PAFs (95% CI)	**0.230** (0.000 to 0.430)				**0.263** (0.000 to 0.586)			

ORs were adjusted (aOR) for age at recruitment (years) ≤50, 51–60, 61–70 or >70 years; age of menarche <12, 12–13, ≥14 or unknown; nulliparity (number of children) none, 1, 2, ≥3 or unknown; breastfeeding time (months) no, <6, ≥ 6 or unknown; energy intake (calories/day) <1500, 1500 to 2000, ≥2000 or unknown; family history of breast cancer no or yes; socioeconomic status low, medium or high; alcohol consumption (past or present g/day) 0, <12, ≥12 or unknown; smoking status never, yes or former; physical activity (METS×hours/week for mean year) 0, <8, 8–16, >16 or unknown; anti-inflammatory drug use never, some or unknown; oral contraceptive treatment never, some or unknown; and oral supplementary hormonal treatment never, some or unknown.

Bold typeface indicates significance at p=0.05.

Erb2+positive human epidermal growth factorPAFspopulation attributable fractions

## Discussion

The burden of breast cancer attributable to excess body fat is likely to be underestimated if assessed with BMI in postmenopausal women, especially in hormone receptor positive tumours. Excess body fat is a well established risk factor for postmenopausal breast cancer,^6^ although why excess body weight is suggested as a protective factor in premenopausal cancer and a risk factor in postmenopausal cancer are not clear.^4 25^ There are several possible explanations, including low oestrogen levels in postmenopausal women.^26 27^ The 2018 Third Expert Report from the World Cancer Research Fund (WCRF) and the Global Cancer Update Programme (CUP) showed that the risk increased by 1.12 for every 5 kg/m^2^ increase in BMI, but with higher risks in Asian populations, hormone receptor positive cancers, and in those receiving hormone replacement therapy.^6^ This positive association in postmenopausal women was consistent when using different indicators of excess body fat (eg, waist circumference or waist-to-hip ratio) or changes in weight throughout life.^9 28^ Our study showed that body fat was associated with an increased risk when measured with a different anthropometric index (ie, CUN-BAE). Although further research is necessary to determine the underlying mechanisms, there are plausible biological explanations linking obesity to carcinogenesis, including associations between obesity and circulating hormone concentrations (eg, insulin, growth factors, oestrogens and adipokines), as well as low grade chronic inflammation.^26 27^

Quantifying the burden of cancer attributable to lifestyle factors is important for preventive programmes and public health decisions. To accurately estimate the burden caused by diseases that can be attributed to a risk factor, a thorough understanding of that risk factor is required. Although we acknowledge that the results of our case–control study cannot establish causal association (because PAF is typically assessed as relative risk), we have tried to provide insight into the impact of differences in the exposure level according to both anthropometric measures. Arnold *et al*^3^ previously estimated that 10% of postmenopausal breast cancers were attributable to a high BMI based on prevalence data from 2002 and GLOBOCAN 2012 to calculate PAFs. In our study, we showed higher estimates of postmenopausal breast cancer cases in Spain attributable to BMI. This difference may be due to the increased prevalence of obesity and the different methods used to calculate risks.^3 29^

Most previous studies that assessed the contribution of excess body fat to breast cancer were carried out using BMI.^4^ It is well known that BMI and its correlation with body fat is affected by race, sex and age, and therefore it tends to underestimate percentage body fat, especially in women and in older persons.^7^ In contrast, after adjusting BMI for age and sex, CUN-BAE showed a better correlation with body fat and also showed better relationship with cardiovascular risk factors and diabetes.^12 15^ Similarly, it has been observed that body fat assessed with BMI might also underestimate the risk of severe cases of influenza compared with body fat assessed with CUN-BAE.^20^ In terms of clinical implementation, CUN-BAE has the simplicity of BMI with improved assessment of body fat, and can be used in primary care with a simple colour scale.^19^

In this study, we found differences in the proportion of postmenopausal breast cancers attributable to body fat when using CUN-BAE (38.0%) compared with BMI (23.0%). These differences were caused by discrepancies in the distribution of the prevalence of overweight and obesity with each method. Moreover, higher ORs estimates were found in the category with a higher percentage of body fat, which suggests that the association between body fat and cancer risk was better stratified using CUN-BAE.

Breast cancer is a heterogeneous disease. As well as differences between premenopausal and postmenopausal diagnoses, various hormone receptors have also been seen to have different aetiologies, and their prognosis and response to treatment may vary. Most breast tumours have hormone receptors, which usually have a better prognosis and response to treatment. Other studies have found an association between BMI and risk of breast cancer exclusively in tumours that expressed a hormone receptor but not in triple negative or Erb2+ tumours.^2^ This is in line with our analysis based on hormone receptors, which showed that differences in attributable fractions according to the method used were observed exclusively in tumours with hormone receptors.

Our results should be interpreted with caution because of the case–control design of the study, although in the MCC-Spain project, population controls were selected and data collection was carried out by trained personnel. BMI was self-reported at the time of the interview for controls and 1 year before diagnosis for cancer cases. Regarding CUN-BAE, one of its limitations is that the formula was calculated from a sedentary convenience sample. The small sample size of cases that did not express hormone receptors is another limitation.

The strengths of the study include its originality; to the best of our knowledge, no previous study has carried out this comparison. Furthermore, it was a multicentre study with population controls and a relatively large sample size, which allowed us to examine specific subtypes of breast tumours, as well as different subgroups (eg, postmenopausal women, different physical activity levels and oral anti-inflammatory drug use). The results obtained with CUN-BAE were independent of its components (sex, age and BMI).^15^

## Conclusions

The results of our study indicate that excess body fat is a significant risk factor for hormone receptor positive breast cancer in postmenopausal women. Our findings suggest that the population impact could be underestimated when using traditional BMI estimates, and that more accurate measures of body fat, such as CUN-BAE, should be considered when estimating the cancer burden attributable to obesity in postmenopausal breast cancer. This information could influence cancer prevention initiatives by highlighting the role of excess body fat in the development of breast cancer and by raising awareness among healthcare professionals and the public.

## Data Availability

Data are available on reasonable request.
